# The existence of adrenal insufficiency in patients with COVID-19 pneumonia

**DOI:** 10.3389/fendo.2024.1337652

**Published:** 2024-07-03

**Authors:** Thachanun Porntharukchareon, Bothamai Dechates, Supamas Sirisreetreerux, Phonthip Therawit, Kriangkrai Tawinprai

**Affiliations:** Department of Medicine, Chulabhorn Hospital, Chulabhorn Royal Academy, Bangkok, Thailand

**Keywords:** adrenal insufficiency, long COVID syndrome, COVID-19 pneumonia, hypocortisolism, low dose synacthen test

## Abstract

**Introduction:**

Infection with SARS-CoV-2 virus may result in long COVID, a syndrome characterized by symptoms such as dyspnea, cardiac abnormalities, cognitive impairment, and fatigue. One potential explanation for these symptoms is hypocortisolism.

**Objective:**

To evaluate the prevalence of hypocortisolism in patients with a history of COVID-19 pneumonia.

**Methods:**

Cross-sectional study of patients who were aged ≥18 years and had a 3-month history of radiography-confirmed COVID-19 pneumonia. Exclusion criteria included current or previous treatment with glucocorticoids and use of an oral contraceptive. Adrenal function was evaluated using a low dose (1ug) corticotropin stimulation test (CST). Serum cortisol levels were measured at 0, 30, and 60 minutes, and baseline plasma ACTH was also measured.

**Results:**

Of the 41 patients enrolled, the median age was 62 years, 17 (42%) were female, and all 41 (100%) had severe pneumonia at baseline. Eleven patients (27%) had hypocortisolism, as evidenced by peak cortisol of less than 402.81 nmol/l after low dose (1 µg) CST. Of these 11 patients, 10 (91%) had secondary hypocortisolism (median ACTH 6.27 pmol/L, range 4.98–9.95 pmol/L) and one had primary hypocortisolism (mean ACTH 32.78 pmol/L). Six of the 11 patients with hypocortisolism (54.5%) reported symptoms of persistent fatigue and 5 (45.5%) required regular glucocorticoid replacement.

**Conclusions:**

Our results suggest that hypocortisolism, predominantly caused by pituitary disruption, may emerge after SARS-CoV-2 infection and should be considered in patients with a history of COVID-19 pneumonia with or without clinical hypocortisolism.

## Highlights

We evaluated the low dose corticotropin stimulation test in patients with history of COVID-19 pneumonia. Our study suggested that participants with history of COVID-19 pneumonia may have adrenal insufficiency predominantly by pituitary disruption. Physicians should be aware of the hypocortisolism in these patients who present with clinical symptoms such as shock, nausea, vomiting, and fatigue.

## Introduction

Coronavirus disease 2019 (COVID-19), caused by severe acute respiratory syndrome coronavirus 2 (SARS-CoV-2), was first identified in Wuhan, China, on December 31, 2019. Since then, the disease has evolved into a global pandemic, fueled by the emergence of multiple SARS-CoV-2 variants. To date, more than 600 million confirmed cases of COVID-19 have been reported worldwide (1). The disease is primarily known for its respiratory symptoms, which range from mild cough to severe acute respiratory distress syndrome (ARDS). However, COVID-19 has also been linked to a variety of multisystemic manifestations, including cardiovascular complications, neurological sequelae, and endocrine dysfunction. In addition, many individuals with COVID-19 experience persistent symptoms, known as “long COVID,” which includes fatigue, headache, dyspnea, and brain fog lasting more than 12 weeks after the initial onset of the illness. Long COVID can involve several organ systems, including the cardiovascular, neurological, gastrointestinal, and endocrine systems (2, 3).

Among the endocrine disorders potentially associated with COVID-19, adrenal insufficiency has gained increasing attention. Adrenal insufficiency can occur in various clinical contexts, including primary adrenal failure (Addison’s disease) and secondary adrenal insufficiency due to hypothalamic-pituitary disorders. The possible link between COVID-19 and adrenal insufficiency is multifaceted. First, the direct cytopathic effects of SARS-CoV-2 on the adrenal glands are a consideration, given the presence of angiotensin-converting enzyme 2 (ACE2) receptors (4), which are the cellular entry points for SARS-CoV-2, in adrenal cortical cells. Second, the systemic inflammatory response triggered by COVID-19, characterized by a cytokine storm and immune dysregulation, may lead to adrenal gland dysfunction through various pathways, including direct tissue damage, disruption of the hypothalamic-pituitary-adrenal (HPA) axis feedback mechanisms, and impairment of adrenal steroidogenesis (5).

During the severe acute respiratory syndrome (SARS) pandemic in 2002–2004, Leow et al. (6) evaluated the function of the hypothalamic–pituitary axis in 61 patients with SARS infection and found evidence of hypocortisolism in 40% of the cohort. The proposed mechanisms included reversible hypophysitis or direct hypothalamic damage caused by the virus. However, data on the prevalence and causes of adrenal insufficiency following SARS-CoV-2 infection are currently limited. Consequently, this study aims to evaluate the prevalence of adrenal insufficiency in adult patients who were discharged from the hospital after being diagnosed with COVID-19 pneumonia and had a 3-month history of COVID-19.

## Materials and methods

### Study design

This was a cross-sectional study that enrolled consecutive patients with a history of COVID-19 pneumonia of 3 months duration at Chulabhorn Hospital, Chulabhorn Royal Academy, Thailand. The study was approved by the Human Research Ethics Committee Chulabhorn Research (Project code 140/2564) and was conducted in accordance with the Declaration of Helsinki and its amendments. All participants provided written informed consent prior to inclusion. The study was registered at the Thai Clinical Trials Registry (TCTR20220606002).

### Participants

Participants were recruited from a COVID-19 cohort ward and acute respiratory unit clinic at Chulabhorn Hospital between March 1, 2022 and April 20, 2022. Eligible patients had a 3-month history of COVID-19 pneumonia and were aged ≥18 years. A diagnosis of COVID-19 was confirmed by real-time reverse-transcriptase polymerase chain reaction of SARS-CoV-2 RNA in nasopharyngeal swab specimens. The severity of COVID-19 was defined as critical, severe, and non-severe according to the World Health Organization (WHO) criteria (7). Critical COVID-19 was defined by as acute respiratory distress syndrome, sepsis shock, sepsis, or the need for life-sustaining therapies such as mechanical ventilation or vasopressor therapy. Severe COVID-19 was defined as one or more of (a) oxygen saturation <90% on room air, (b) signs of pneumonia, or (c) signs of severe respiratory distress. Non-severe COVID-19 was defined as the absence of any criteria for severe or critical COVID-19. Pneumonia was confirmed by imaging with either a chest radiograph or computed tomography (CT) of the chest. Among the exclusion criteria were comorbidity with pituitary disease or adrenal disease; unstable clinical condition; chronic kidney disease (creatinine >1.5 mg/dL); severe hepatitis (alanine aminotransferase >1.5× upper limit of normal); pregnancy or plans to become pregnant; and concurrent use of oral contraceptive pills, steroids (oral, inhaled, topical, or intra-articular), and other medications known to affect cortisol-binding globulin (including oral estrogens). Suitable subjects identified from a review of case notes were contacted in person or via the telephone.

### Image analysis

COVID-19 pneumonia was confirmed by chest radiography or CT scan. CT scans were performed on the first and second day after COVID-19 diagnosis. The severity of pneumonia on CT was scored according to the COVID-19 Reporting And Data System (CO-RADS) classification using lobar-based assessment. In brief, each of the five lung lobes was subjectively scored from 0 to 5 (0, no involvement; 1, <5% involvement; 2, 6–25% involvement; 3, 26–50% involvement; 4, 51–75% involvement; 5, ≥76% involvement). The total score was the sum of the individual lobar scores and ranged from a minimum of 0 to a maximum of 25. Total scores of <7, 8–17, and 18–25 were classified as mild, moderate, and severe pneumonia, respectively (8).

### Study protocol

Blood samples were collected at baseline for measurement of plasma ACTH and serum cortisol, free thyroxine (FT4), thyroid-stimulating hormone (TSH), anti-thyroglobulin (anti-Tg), and anti-thyroid peroxidase (anti-TPO). Corticotropin stimulation test (CST) were performed as described below. Participants with hypocortisolism were evaluated for potential causes other than SARS-CoV-2 infection by magnetic resonance imaging (MRI) of the pituitary gland or an adrenal CT protocol as appropriate.

### Corticotropin stimulation test

Low-dose (1 µg) CST were performed between 8 am and 10 am. An intravenous catheter was inserted into an antecubital vein and 0.4 mL (1 µg) of a synthetic ACTH 1–24 solution (Synacthen^®^, Novartis, Chippenham, UK) in 0.9% sodium chloride was administered through the catheter, followed by flushing with 15 mL of 0.9% sodium chloride. Blood samples were collected immediately before Synacthen injection to determine baseline ACTH and cortisol, and at 30 min and 60 min after Synacthen injection to determine the cortisol response to stimulation. A diagnosis of hypocortisolism was defined as a peak cortisol level of <402.81 nmol/L based on the new criteria for the Roche Elecsys Cortisol Generation II assay (Roche Diagnostic, Mannheim, Germany) (9, 10). Primary hypocortisolism was defined as a baseline plasma ACTH level >2× the upper limit of the reference range (11).

### Laboratory investigations

Serum cortisol and plasma ACTH were measured using automated electrochemiluminescence immunoassays (Roche Elecsys Cortisol Generation II assay and Roche Elecsys ACTH assay; Roche Diagnostic, Mannheim, Germany). For plasma ACTH measurement, blood was collected in EDTA tubes and immediately transferred to the laboratory. FT4, TSH, anti-Tg, and anti-TPO were also measured using automated electrochemiluminescence immunoassays on the Roche Elecsys system. Reference ranges were 2.86–13.86 pmol/L for plasma ACTH, 11.97–21.88 pmol/L for FT4, and 0.27–4.2 mIU/L for TSH.

### Statistical analysis

The sample size calculation was based on an infinite population proportion formula (12) and previous analysis of the prevalence of hypocortisolism in SARS-CoV-2 patients (6). Data analysis was conducted using STATA/SE version 16.1 (StataCorp LP, College Station, TX, USA). Continuous data are presented as mean ± standard deviation (SD) or the median with interquartile range (IQR) as appropriate. Normally distributed continuous data were compared using a Student’s t-test for two groups or by one-way analysis of variance for three or more groups. Non-normally distributed continuous data were compared using the Mann–Whitney U test for two groups or the Kruskal–Wallis test with Dunn’s *post hoc* test for three or more groups. Categorical data were compared using a Chi-squared test. An analysis of weight change was conducted using analysis of covariance (ANCOVA) to compare participants with hypocortisolism to those without hypocortisolism at both baseline and the three-month follow-up. The relationship between two continuous variables was determined using Pearson’s correlation. The α level for statistical significance was set at 0.05.

## Results

### Participants

**Figure 1:**
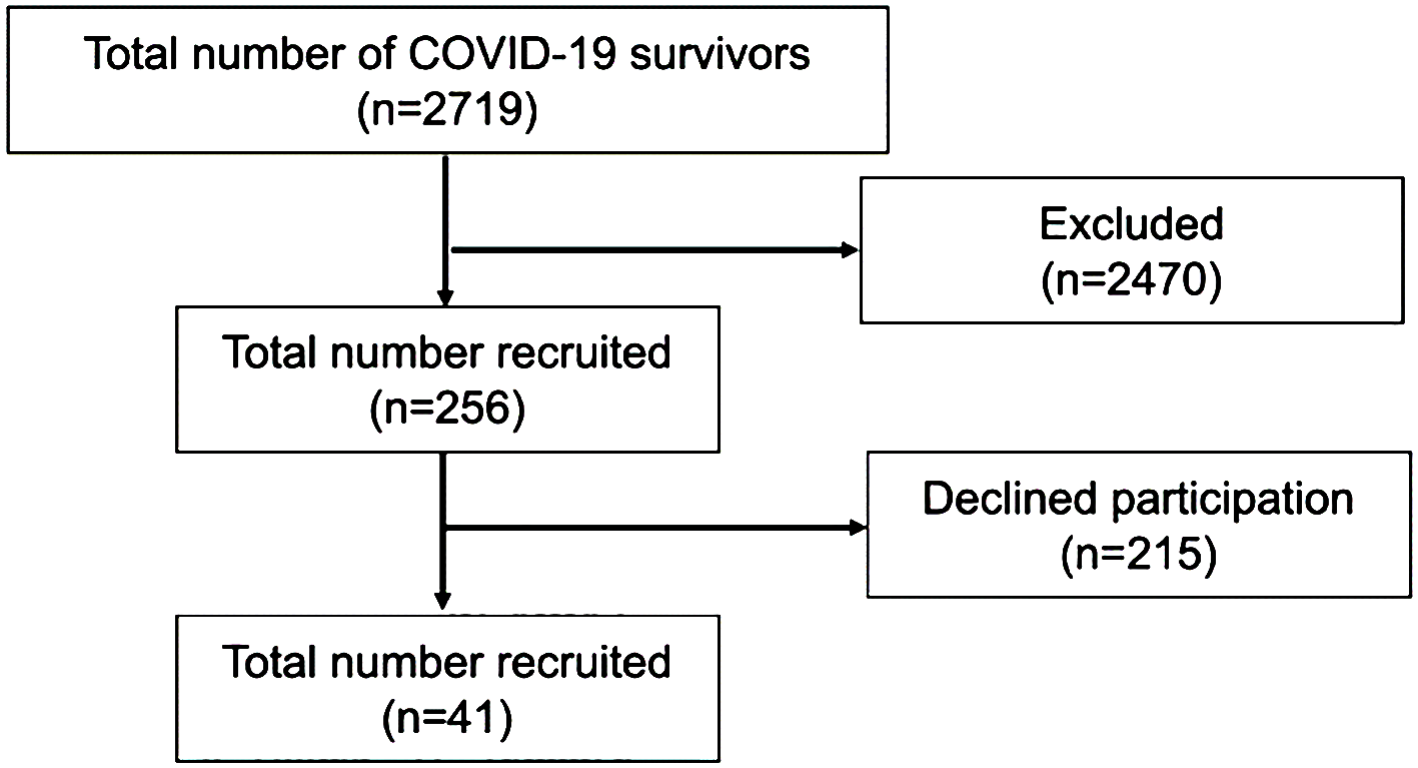
Study participation.

Medical records of 2719 patients seen at our hospital between March 1, 2022 and April 20, 2022 were reviewed. A total of 2463 patients were excluded (182 patients <18 years of age, 2 patients were deceased, and 2279 patients had no documentation of pneumonia by radiography). The remaining 256 participants were contacted in person or by telephone and 215 declined to participate. Finally, a total of 41 patients were enrolled in the study (**Figure 1**).

The study cohort comprised 41 patients, with a mean (SD) age of 57.1 (13.7) years. Among them, 17 (41%) were female. All of the patients had severe COVID-19 pneumonia at baseline based on the WHO criteria (6). Thirty-seven patients underwent chest CT; based on the CO-RADS system of pneumonia classification (7), 36 of the 37 patients (87.8%) had mild pneumonia and 1 patient (2.4%) had moderate pneumonia. None of the patients developed acute respiratory failure. Most patients were obese (29/41; 70.7%) and the mean body mass index (BMI) of the full cohort was 28.85 kg/m^2^. Fifteen patients (36.7%) received dexamethasone treatment for COVID-19. Of the fifteen COVID-19 patients who received glucocorticoid treatment, fourteen were administered dexamethasone at a dosage of 6 milligrams per day for a duration of 10 days. One patient received dexamethasone at a dosage of 18 milligrams per day for 5 days, followed by 6 milligrams per day for an additional 5 days. Other baseline characteristics of the study participants are shown in **Table 1**.

**Table 1:** Baseline characteristics of the 41 study participants.

Baseline characteristics	
Sex (female) (number, %)	17 (41.5%)
Age (years) (mean and SD)	57.1 (13.7)
BMI (kg/m^2^) (mean and SD)	29.4 (6.4)
Obesity (number, %)	29 (70.7%)
Diabetes mellitus (number, %)	13 (31.7%)
Hypertension (number, %)	23 (56.1%)
Cardiovascular disease (number, %)	1 (2.4%)
COVID-19 severity (WHO category) (number, %)	
Critical	0
Severe	41 (100%)
Non-severe	0
Symptoms (number, %)	
Fever	14 (34.1%)
Upper respiratory tract involvement	34 (82.9%)
Diarrhea	7 (17.1%)
Dyspnea	3 (7.3%)
Fatigue	8 (19.5%)
Rash	2 (4.9%)
Treatment (number, %)	
Dexamethasone	15 (36.6%)
Favipiravir	38 (92.7%)
Sotrovimab	3 (7.3%)
Convalescent plasma	6 (14.6%)

BMI, body mass index; CRP, C-reactive protein; IQR, interquartile range; WHO, World Health Organization.

**Figure 2:**
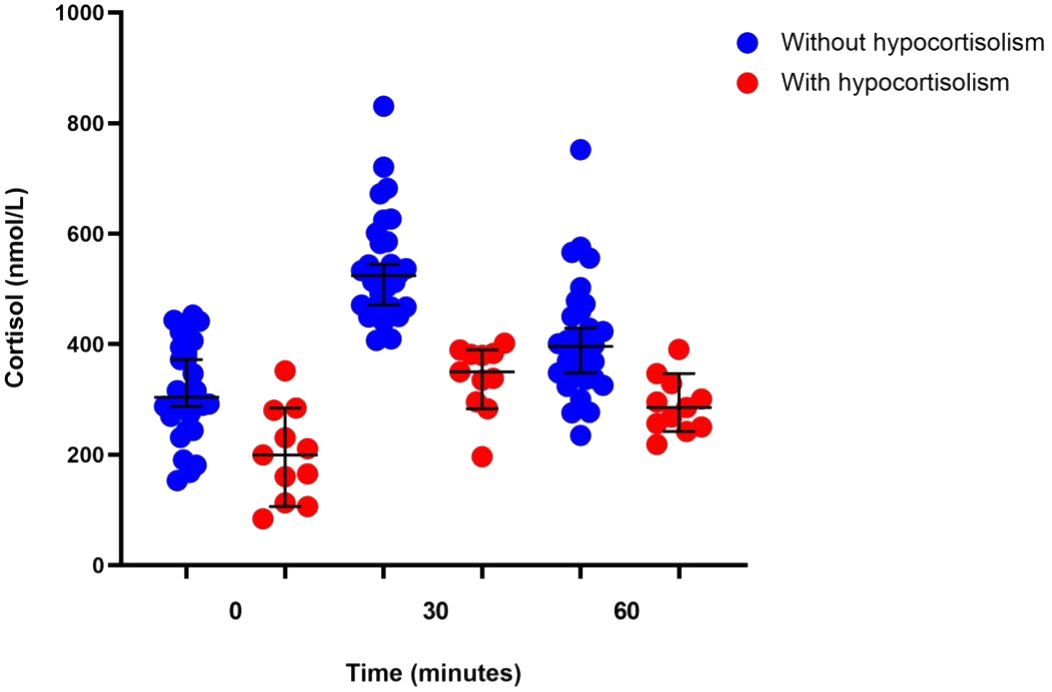
Corticotropin stimulation test results in patients with (n = 11) or without (n = 30) hypocortisolism.

Eleven (27%) of the 41 participants showed evidence of hypocortisolism in the CST (**Figure 2**). The mean baseline cortisol level was 198.92 ± 83.87 nmol/L, and 3 patients had baseline cortisol levels <137.95 nmol/L. Of the 11 patients with hypocortisolism, 10 (90.9%) were diagnosed with central hypocortisolism based on low-to-normal ACTH levels (median ACTH 6.27 pmol/L, IQR 4.98–9.95). The remaining patient had a plasma ACTH level of 32.78 pmol/L and was diagnosed with primary hypocortisolism. Six of the 11 patients (54.50%) reported persistent fatigue after resolution of the acute infection. Participants with hypocortisolism had nonsignificantly greater reduction in body weight compared to those without hypocortisolism (-2.00 kg, 95% CI -4.42 to 0.42 vs. 0.21 kg, 95% CI -0.37 to 0.78, p = 0.079). Only 5 of the 11 patients received corticosteroid therapy for the treatment of COVID-19 (**Table 2**). Six of the 10 patients with secondary hypocortisolism underwent MRI of the pituitary and no abnormal lesions were found. Four patients did not perform MRI imaging due to personal reason. The patient with primary hypocortisolism underwent an adrenal CT protocol and had normal bilateral adrenal glands.

**Table 2:** Clinical characteristics and laboratory values of patients with hypocortisolism (n=11).

Patient ID	Age (years)	Sex	CT score^a^	Treatment	Cumulative dose of Dex (mg)	Persistent fatigue	Cortisol level in CST (nmol/L)			ACTH (pmol/L)
							Baseline	30 min	60 min	
No. 1	73	M	2	Fa, C	None	Yes	199.5	383.5	256.6	12.80
No. 2^b^	29	M	2	Fa, C	None	No	210.8	295.8	218.8	19.07
No. 3^b^	33	M	NA	Fa	None	Yes	106.5	196.7	328.6	6.25
No. 4	63	M	4	S	None	No	284.5	389.8	268.2	32.78
No. 5^b^	51	M	NA	Fa	None	No	230.9	334.9	300.7	6.29
No. 6	46	F	1	Fa, Dex	60	Yes	351.8	381.6	295.2	6.05
No. 7	67	F	2	Fa, Dex	60	Yes	280.6	283.1	390.7	3.70
No. 8	21	M	2	Fa, C	None	Yes	84.1	402.0	250.2	5.41
No. 9	34	F	NA	Fa, Dex	60	No	165.3	379.9	285.3	9.00
No. 10	67	F	1	Fa, Dex	60	No	160.6	350.1	242.2	6.64
No. 11	71	F	6	Fa, Dex	60	Yes	112.8	338.3	346.8	2.82

ACTH, adrenocorticotropic hormone; C, convalescent plasma; CST, corticotropin stimulation test; CT, computer tomography; Dex, dexamethasone; F, female; Fa, favipiravir; ID, identification number; mg, milligrams; M, male; NA, not available; No, number; S, sotrovimab.

^a^CO-RADS classification.

^b^Patients who still had hypocortisolism when the cut-off for low dose corticotropin stimulation test was 345 nmol/L.

In addition to the varied cut-off levels for hypocortisolism, we implemented a more stringent cut-off value of 345 nmol/L for the low-dose synacthen test to ensure robustness in our analysis. Despite this stricter criterion, three patients (7%) still exhibited hypocortisolism. Detailed characteristics and laboratory test results for patients numbered 2, 3, and 5 are provided in **Table 2**.

Two of the 41 patients (5%) had abnormal thyroid function tests; one had iatrogenic thyrotoxicosis, and positive autoimmune thyroid antibodies. She was being treated with levothyroxine suppressive therapy for papillary thyroid cancer, and the second had subclinical hypothyroidism (thyroid-stimulating hormone 5.4 µIU/mL). Five patients (12%) were positive for anti-thyroid peroxidase or anti-thyroglobulin antibodies with normal thyroid function test. Further clinicopathological details of the study participants is provided in **Supplementary Table 1**

There is no statistical significance, but a trend towards more fatigue symptoms in participants with adrenal insufficiency compared to those without hypocortisolism. The odds ratio for fatigue was 3.19 (95% CI, 0.70 to 14.56), with a p-value of 0.135 in participants with hypocortisolism (**Table 3**). Furthermore, none of the participants reported symptoms of brain fog, headache, memory problems, or muscle pain. We also performed logistic regression analysis to determine the risk factors associated with hypocortisolism in the subsets of patients with (n = 11) or without (n = 30) hypocortisolism. Increased BMI (adjusted P = 0.019) was the only significant risk factor for hypocortisolism. Age, sex, treatment modality, glucocorticoid usage, history of COVID-19 vaccination, and disease severity were not associated with hypocortisolism (**Table 3**).

**Table 3:** Evaluation of risk factors for hypocortisolism by logistic regression.

Characteristic	Patients with hypocortisolism (n=11)	Patients without hypocortisolism (n=30)	Crude OR (95% CI)	P value	Adjusted OR (95% CI)	P value
Sex, female	5 (45.5%)	12 (40.0%)	1.25 (0.31–5.04)	0.754		
Age, years (IQR)	51 (33–67)	62 (59–65)	0.95 (0.91–1.00)	0.067	1.04 (0.96–1.12)	0.380
BMI, kg/m^2^ (IQR)	34.96 (29.86–37.52)	26.3 (24.61–30.0)	1.23 (1.05–1.43)	**0.009**	1.27 (1.04–1.54)	**0.019**
Comorbidities						
Obesity	10 (90.9%)	19 (63.3%)	5.79 (0.65 –51.50)	0.115		
Diabetes mellitus	5 (45.5%)	8 (26.7%)	2.29 (0.54–9.64)	0.258		
Hypertension	3 (27.3%)	20 (67.7%)	0.19 (0.04–0.86)	**0.032**	0.17 (0.02–1.23)	0.079
CT score (CO-RADS)						
<7	7 (72.7%)	28 (96.6%)	4.00 (0.22–72.18)	0.348		
8–17	1 (12.5%)	1 (3.4%)				
≥18	0	0				
Laboratory investigations						
SARS-CoV-2 antibody (mean ± SD BAU/mL) **^a^**	5475.57 ± 4646.10	17425.10 ± 7900.03	0.99 (0.99–1.00)	0.257		
Serum CRP (mg/L)	6.09 (3.33–12.83)	9.68 (4.60–12.76)	1.02 (0.98–1.05)	0.426		
Persistent fatigue	6 (60.0%)	8 (32.0%)	3.19 (0.70–14.56)	0.135		
Treatment						
Dexamethasone	5 (45.5%)	10 (33.3%)	1.67 (0.41–6.82)	0.477		
Favipiravir	10 (90.9%)	28 (93.3%)	0.71 (0.06–8.76)	0.792		
Sotrovimab	1 (9.1%)	2 (6.7%)	1.40 (0.11–17.17)	0.792		
Convalescent plasma	3 (27.3%)	3 (10.0%)	3.38 (0.57–20.10)	0.181		
COVID–19 vaccine^b^						
CoronaVac	2 (20.0%)	7 (23.3)	0.82 (0.14–4.80)	0.827		
BBIBP-CorV	2 (20.0%)	4 (13.3%)	1.63 (0.25–10.58)	0.611		
ChAdOx1-S	8 (80.0%)	24 (80.0%)	1.00 (0.17–5.98)	1.000		
BNT162b2	5 (50.0%)	22 (73.3%)	0.36 (0.08–1.60)	0.180		
mRNA-1273	2 (20.0%)	2 (6.7%)	3.50 (0.42–28.91	0.245		

BAU, binding antibody unit; BMI, body mass index; CI, confidence intervals; CRP, C-reactive protein; CO-RADS, COVID-19 Reporting And Data System; CT, computed tomography; IQR, interquartile range; OR, odds ratio; SD, standard deviation.

**^a^** Based on the WHO international standard for anti-SARS-CoV-2 Ig. One Elecsys-S unit = 0.972 × binding antibody unit.

^b^10 of the 11 patients with hypocortisolism had documentation of COVID-19 vaccination. The values in bold denote statistical significance at P < 0.05.

## Discussion

The results of our study suggest that hypocortisolism is a common complication in patients with COVID-19 pneumonia, with most cases in our cohort (10 of 11 patients) manifesting as central hypocortisolism. Additionally, a significant proportion of these patients in (55%) reported clinical symptoms of long COVID, including fatigue, insomnia, and dyspnea.

The underlying mechanism of hypocortisolism in COVID-19 patients is likely to involve the ACE2 receptor, the major functional receptor for infection by both SARS-CoV and SARS-CoV-2. Indeed, ACE2 has been detected in many human tissues, including endocrine glands such as the adrenal and pituitary glands (13–16). ACE2 receptors mediate viral entry in concert with S glycoprotein priming by the host cell transmembrane serine protease 2 (13, 14).

Many studies have reported cases of pituitary disruption after SARS-CoV-2 infection, including central hypocortisolism, central diabetes insipidus, hypothalamic hypogonadism, lymphocytic hypophysitis, and pituitary apoplexy. Primary hypocortisolism has also been reported in COVID-19 patients. Details of previously reported cases of pituitary dysfunction and primary hypocortisolism occurring more than 2 weeks after SARS-CoV-2 infection are shown in **Table 4** (17–23, 25, 27, 28). The findings from those studies are consistent with our own results and support the conclusion that pituitary and adrenal function may be affected by SARS-CoV-2 infection.

**Table 4:** Literature cases of hypothalamic–pituitary dysfunction and primary adrenal insufficiency occurring more than 2 weeks after SARS-CoV-2 infection.

Study (ref. no.)	Patient age/sex	Time to onset after infection	Presentation	Results of investigations	Diagnosis
Secondary adrenal insufficiency					
Kenya et al. (17)	23/F	1 month	Fatigue, nausea, vomiting	• Basal cortisol 226.24 nmol/L; ACTH 1.08 pmol/L • hypocortisolism was confirmed with insulin tolerance test. • MRI pituitary: normal.	Secondary adrenal insufficiency
Central diabetes insipidus					
Sheikh et al. (18)	28/M	1 month	Polyuria, polydipsia, increased thirst	• 24-hr urine volume 7 L. • Serum sodium 153 mmol/L; paired serum and urine osmolality 300 and 93 mOsm/kg, respectively; urine sodium 16 mOsm/kg. • MRI brain: normal.	DI with concomitant myocarditis
Yavari et al. (19)	54/F	6 weeks	Thirst, polyuria, polydipsia	• 24-hr urine volume 13.3 L. • Serum sodium 144 mmol/L; paired serum and urine osmolality 298 and 164 mOsm/kg, respectively. • Urine osmolality 810 mOm/kg after intravenous desmopressin administration test. • MRI pituitary: normal.	CDI
Misgar et al. (20)	60/F	8 weeks	Polyuria	• 24-hr urinary volume 6 L. • Serum sodium 152 mmol/L; paired serum and urine osmolality 300 and 177 mOsm/kg, respectively. • MRI pituitary: enlarged pituitary with absent posterior pituitary bright spot on T1-weighted images; thickening of pituitary stalk.	CDI
Pituitary apoplexy					
Liew et al. (21)	75/M	1 month	Sudden onset severe frontal headache	• FT4 6.9 pmol/L (reference range: 10.5–24.5), TSH 0.1 mU/L (0.27–4.2), cortisol 57 nmol/L (133–537), testosterone <0.5 nmol/L (6.7–25.7), LH <1.0 U/L (1.7–8.6). • MRI: pituitary macroadenoma with recent hemorrhage.	Pituitary apoplexy with hypopituitarism
Hypothalamic hypogonadism					
Soejima et al. (22)	36/M	99 days	Insomnia, headache, dysgeusia, alopecia	• Free testosterone 19.09 pmol/L (22.56–61.42), FSH 4.2 IU/L (1.3–17), LH 3.0 IU/L (0.52–7.8). • MRI pituitary: partially empty sella.	Hypothalamic hypogonadism
Facondo et al. (17)	36/F	6 months	Secondary amenorrhea	• Estradiol <91.77 pmol/L (91.77–921.42), FSH 3.85 IU/L (3.0–8.0), LH 0.29 IU/L (1.8–11.78), TSH 1.71 mIU/L (0.27–4.2). • GnRH analog test: normal response. • TRH test: delayed response • MRI brain and pituitary: uncertain pituitary microadenoma 3 mm.	Hypothalamic amenorrhea
Lymphocytic hypophysitis					
Joshi et al. (23)	18/F	3 weeks	Acute onset headache	• Hormonal workup: within normal limits. • MRI brain: diffuse thickening and enlargement of the infundibulum with homogenous contrast enhancement.	Lymphocytic hypophysitis
Gorbova et al. (24)^a^	35/F	2 months	Symptoms of hypopituitarism	• Hormonal workup: hypothyroidism, hypocorticism, hypogonadism. • MRI: hypophysitis.	Hypophysitis and reversible hypopituitarism
Primary adrenal insufficiency					
Eskandari et al. (25)	18/M	2 weeks	Severe weakness, acute chest pain, hypotension	• Serum sodium 129 mmol/L; 8 am cortisol 38.63 nmol/L; ACTH >396 pmol/L. • Cortisol levels at baseline and 60 min after 250 µg ACTH stimulation test were 49.67 and 281.42 nmol/L, respectively. • Anti-21-hydroxylase antibody: positive.	Autoimmune PAI and myocarditis
Machado et al. (26)	46/F	3 weeks	Malaise, nausea, vomiting, hyperpigmentation, postural hypotension	• CT abdomen: adrenal infarction.	PAI with bilateral adrenal infarction
Sánchez et al. (27)	65/F	5 months	Abdominal pain nausea, vomiting, weight loss	• Serum sodium 117 mmol/L • Cortisol at baseline 71.73 nmol/L; ACTH at baseline 427.68 pmol/L • Cortisol at baseline, 30, and 60 min in 250 µg ACTH stimulation test were 63.46, 80.01, and 71.73 nmol/L, respectively • Anti-21-hydroxylase antibody: present. • CT abdomen: unremarkable.	Autoimmune PAI

ACTH, adrenocorticotropic hormone; CDI, central diabetes insipidus; CT, computer tomography; DI, diabetes insipidus; F, female; FSH, follicle-stimulating hormone; FT4, Free thyroxine; GnRH, gonadotropin-releasing hormone; hr, hour(s); LH, luteinizing hormone; M, male; MRI, magnetic resonance imaging; min, minutes; no., number; PAI, primary adrenal insufficiency; TRH, thyrotropin-stimulating hormone; ref, reference; TSH, thyroid-stimulating hormone.

^a^Only the abstract was available in English.

In the present study, high BMI patients was significantly associated with an increased risk of hypocortisolism. This could be explained by high ACE2 expression in adipose tissue resulting in an increased viral burden, thereby increasing virus-associated damage to the endocrine glands (16). Other potential mechanisms are an increased proinflammatory phenotype associated with metabolic dysfunction and dysregulation of the renin–angiotensin pathway (29). Obesity is also an important risk factor for increased severity of COVID-19.

In our study, most of the patients with hypocortisolism exhibited non-critical COVID-19 disease severity. During the 6-month follow-up period, one patient received a daily dose of prednisolone at 5 mg, while the remaining patients received corticosteroids as needed, primarily during illness episodes.

Our study results differ from those of Clarke et al. (30) who previously found no evidence of hypocortisolism in patients with COVID-19 (30). However, the two studies differed in that we performed the CST using a low dose (1 µg) of CST whereas Clarke et al. used the standard CST (250 µg ACTH), which may have failed to detect patients with mild or early onset hypocortisolism (31). The results of our study are similar to those of Urhan et al. (32) except that the frequency of patients with hypocortisolism was higher in our study (27% vs 16.2%). This may be due to differences between the two studies in the severity and duration of COVID-19, especially because all of our study participants had severe COVID-19 pneumonia. Another difference between the two studies was that we evaluated adrenal function within 3 months of SARS-CoV-2 infection compared with the study of patients at 3–7 months post-infection by Urhan et al. (32).

A strength of our study lies in the utilization of a low cut-off value for serum cortisol (402.8 nmol/L) to define hypocortisolism, a measure that likely contributed to a decreased false-positive rate. Traditionally, the practical cut-off values for diagnosing adrenal insufficiency following standard and low-dose CST has been set at 500 nmol/L and has been widely accepted for an extended period (11, 33, 34). In our study, we adopted a similar approach by evaluating adrenal function using a low-dose CST, a strategy aimed at mitigating false-negative results. However, recent investigations, such as those conducted by Javorsky BR et al. utilizing the Elecsys^®^ Cortisol II assay, have identified a lower cut-off value of 402.8 nmol/L for diagnosing hypocortisolism following a standard dose CST (9). Furthermore, we conducted imaging studies to explore other potential etiologies of adrenal insufficiency in the majority of the 11 study participants diagnosed with hypocortisolism.

However, it is important to acknowledge certain limitations in our study. A significant limitation is the absence of a widely accepted standard cut-off value for the low-dose CST specifically for the Roche Elecsys Cortisol Generation II assay. Although we used a lower cut-off value than previously established, the cut-off value for low-dose CST should ideally be lower than that for standard CST. Accurate diagnosis of hypocortisolism relies heavily on precise cortisol cut-off values, which are inherently assay and protocol-dependent. In our study, we set the peak cortisol level cut-off at 30 or 60 minutes of the low-dose CST at 402.8 nmol/L, based on recommendations from Javorsky BR et al. and Mongioi et al. (9, 10). However, it is worth noting that this approach may potentially lead to an overestimation of hypocortisolism diagnoses.

To address this concern, we explored alternative cut-off values suggested by Karaca Z et al. (35), which utilized different assays but employed low-dose CST. This study measured serum cortisol levels by radioimmunoassay (RIA) method and established a cut-off value of 345 nmol/L. Reassessment of our data using this cut-off revealed that three out of 41 participants (7%) remained diagnosed with hypercortisolism. Notably, all three of these participants did not receive corticosteroid treatment during COVID-19 management. As a result, eight participants exhibited cortisol levels falling within a grey zone for the diagnosis of hypocortisolism.

Due to the absence of a defined standard cut-off value for the low-dose CST by the Elecsys^®^ Cortisol II assay, it is conceivable that some of these participants may indeed suffer from hypocortisolism. This result underscores the urgency of determining the appropriate cut-off value for the low-dose CST using the Elecsys^®^ Cortisol II assay. A higher cut-off value provides more sensitivity but lower specificity, and vice versa. Therefore, further studies are urgently needed to establish an optimal cut-off value that balances sensitivity and specificity for accurate diagnosis of hypocortisolism. In addition, the sample size was small, and the study was conducted at a single center, both of which might impede the generalizability of our findings. Four participants with secondary adrenal insufficiency did not undergo MRI of the pituitary gland, which could have identified potential pituitary abnormalities. This limitation was due to the participants’ decision to decline the procedure when invited. Another limitation involves the potential for selection bias because participants may have been more inclined to join the study based on their concern about their post-COVID condition or the presence of symptoms resembling adrenal insufficiency. We made efforts to minimize this bias through formal consecutive invitations to all eligible patients, but it may still exist. Furthermore, there was no assessment of baseline pituitary (ACTH)-adrenal (cortisol) function in participants before their COVID-19 diagnosis or hospitalization. Determining whether participants had impaired function is challenging, especially during the critical phase of severe COVID-19 at presentation, which may lead to false-negative test outcomes. However, we addressed this limitation by excluding patients with a history of pituitary or adrenal disease and those concurrently using medications known to affect pituitary-adrenal function. Additionally, all study participants had severe COVID-19 pneumonia, and the results may not accurately represent patients with milder forms of the disease or critically ill patients.

Overall, the results of this study highlight the importance of monitoring for endocrine complications in patients with a history of SARS-CoV-2 infection. Further research will be needed to fully understand the underlying mechanisms of hypocortisolism following SARS-CoV-2 infection, and additional studies with larger sample sizes are needed to confirm the findings of this study and to better understand the prevalence and mechanisms of hypocortisolism in patients with COVID-19 pneumonia.

We conclude that patients with a history of COVID-19 pneumonia who present with clinical symptoms such as shock, nausea, vomiting, and fatigue should have hypocortisolism included in the differential diagnosis. These patients should also be followed over the long term to understand the persistence of adrenal insufficiency and its recovery rate. Finally, all patients who suffer from long COVID syndrome might benefit from an analysis of hypothalamic–pituitary axis function.

## Data availability statement

The original contributions presented in the study are included in the article/**Supplementary Materials**, further inquiries can be directed to the corresponding author/s.

## Ethics statement

The studies involving humans were approved by Human Research Ethics Committee Chulabhorn Research Institute. The studies were conducted in accordance with the local legislation and institutional requirements. The participants provided their written informed consent to participate in this study.

## Author contributions

TP: Conceptualization, Investigation, Methodology, Writing – original draft, Writing – review & editing. BD: Investigation, Writing – original draft. SS: Investigation, Writing – review & editing. PT: Investigation, Writing – review & editing. KT: Conceptualization, Writing – review & editing.
