# Supplementary material for: The existence of adrenal insufficiency in patients with COVID-19 pneumonia
Source: Front Endocrinol (Lausanne). 2024 Jul 3;15:1337652. doi: 10.3389/fendo.2024.1337652 (PMC11251879; doi:10.3389/fendo.2024.1337652)
Supplement: Supplementary file 1 [file DataSheet_1.pdf]

Supplemental Data

Supplementary Table 1. Clinical characteristics and laboratory values of patients with abnormal thyroid function test or positive for autoimmune thyroid antibody (n=7).

Patient ID	Age (years)	Sex	CT score[a]	Treatment	AI	FT4 (pmol/L)[b]	TSH (uIU/mL)[b]	Anti-TPO (IU/mL)[b]	Anti-Tg (IU/mL)[b]	Notes
No. 6	46	F	1	Fa, Dex	Yes	18.15	0.962	157	233	-
No. 22	64	F	2	Fa, Dex	No	15.32	2.04	78.4	51	-
No. 23	59	F	12	Fa, C	No	38.87	0.013	112	961	PTC on LT4 suppressive therapy
No. 24	64	F	1	Fa, Dex	No	14.03	1.67	1110	311	-
No. 28	66	F	5	Fa, Dex	No	14.93	1.07	215	542	-
No. 33	75	M	1	Fa	No	13.51	3.1	>3000	1975	-
No. 41	62	F	1	Fa	No	11.97	5.25	13.6	18	-

Abbreviations: AI, Adrenal insufficiency; Anti-TPO, Anti-thyroid peroxidase; Anti-Tg, Anti-thyroglobulin; C, convalescent plasma; CT, computer tomography; Dex, dexamethasone; F, female; Fa, favipiravir; FT4, Free thyroxine; ID, identification number; LT4, levothyroxine; M, male; No, number; PTC, Papillary thyroid carcinoma; TSH, Thyroid stimulating hormone.

[a] CO-RADS classification.

[b] Reference ranges: FT4 11.97–21.88 pmol/L, TSH 0.27–4.2 uIU/mL, anti-TPO <34 IU/mL, anti-Tg <115 IU/m.
