# Supplementary material for: The existence of adrenal insufficiency in patients with COVID-19 pneumonia
Source: Front Endocrinol (Lausanne). 2024 Jul 3;15:1337652. doi: 10.3389/fendo.2024.1337652 (PMC11251879; doi:10.3389/fendo.2024.1337652)
Supplement: Supplementary file 2 [file DataSheet_2.pdf]

**Supplement 2:** Studies using low dose (1ug) corticotropin stimulation test

| Author, Years | Objective | n | Cortisol assay | Cut-off |
|---|---|---|---|---|
| Soule et al. 2000 (1) | Compare to metyrapone test in patients with suspected pituitary disease. | 86 | CS180 analyser (Chiron Diagnostics, Halstead, UK) | 414 nmol/L (sensitivity 50%, specificity 100%)<br>600 nmol/L (sensitivity 83%, specificity 58%) |
| Karaca Z et al. 2011 (2) | Comparison of low and standard dose ACTH and glucagon stimulation tests in the evaluation of hypothalamo-pituitary-adrenal axis in healthy adult. | 55 | radioimmuno- assay (RIA) method with DSL-2100 (Texas, USA) | 344 nmol/L |

| Author, Years | Objective | n | Cortisol assay | Cut-off |
| --- | --- | --- | --- | --- |
| Dekkers OM et al. 2011 (3) | Comparison cortisol response in LDST and HDST in patients with suspeceted adrenal insufficiency | 207 | fluorescence immunoassay on a TDx (Abbott Laboratories) | 550 nmol/L |
| Lekkakou L et al. 2013 (4) | Effect of gender and age on HPA reactivity | 61 | immunochemiluminescence method (ICMA) using the ADVIA Centaur CP Immunoassay System | 497 nmol/L |
| Anantharaman R et al. 2013 (5) | Evaluate the 1 ug cosyntropin test in normal individulas | 49 | automated Immuno chemiluminometric Access2 assay system (Beckmann- Coulter Gallaway, Ireland). | 455 nmol/L |
| Mak IYF et al. 2017 (6) | Cortisol response after LDST | 217 | Chemiluminescent microparticle immunoassay using the Abbott Architect i2000SR system (Abbott Laboratories, Abbott Park, IL) | 376 nmol/L |

| **Author, Years** | **Objective** | **n** | **Cortisol assay** | **Cut-off** |
|---|---|---|---|---|
| Kline GA et al. 2017 (7) | Cortisol response after LDST | 11 | Elecsys Cortisol II | 350 nmol/L (sensitivity, specificity 97%) |
| Mongioì LM. 2019 (8) | Cortisol cut-off for diagnosis | 103 | enzyme-linked immunocolorimetric assay | 500 nmol/L (sensitivity 100%, specificity 67.3%)<br>401.5 nmol/L (sensitivity 100%, specificity 93.9%) |
| Grassi G et al. 2020 (9) | Cortisol cut-off using compare Roche cortisol1 and Roche cortisol 2 | 30 | Elecsys Cortisol II | 368 nmol/L |

**Reference**

1. Soule S, Van Zyl Smit C, Parolis G, Attenborough S, Peter D, Kinvig S, et al. The low dose ACTH stimulation test is less sensitive than the overnight metyrapone test for the diagnosis of secondary hypoadrenalism. Clin Endocrinol (Oxf). 2000;53(2):221-7.
2. Karaca Z, Lale A, Tanriverdi F, Kula M, Unluhizarci K, Kelestimur F. The comparison of low and standard dose ACTH and glucagon stimulation tests in the evaluation of hypothalamo-pituitary-adrenal axis in healthy adults. Pituitary. 2011;14(2):134-40.
3. Dekkers OM, Timmermans JM, Smit JW, Romijn JA, Pereira AM. Comparison of the cortisol responses to testing with two doses of ACTH in patients with suspected adrenal insufficiency. Eur J Endocrinol. 2011;164(1):83-7.
4. Lekkakou L, Tzanela M, Lymberi M, Consoulas C, Tsagarakis S, Koutsilieris M. Effects of gender and age on hypothalamic-pituitary-adrenal reactivity after pharmacological challenge with low-dose 1-mug ACTH test: a prospective study in healthy adults. Clin Endocrinol (Oxf). 2013;79(5):683-8.
5. Anantharaman R, Menezes G, Yusuf R, Ganapathi B, Ayyar SV, Srinivasan R. The 1 mug cosyntropin test in normal individuals: A reappraisal. Indian J Endocrinol Metab. 2013;17(4):693-6.
6. Mak IYF, Au Yeung BYT, Ng YW, Choi CH, Iu HYP, Shek CC, et al. Salivary Cortisol and Cortisone After Low-Dose Corticotropin Stimulation in the Diagnosis of Adrenal Insufficiency. J Endocr Soc. 2017;1(2):96-108.
7. Kline GA, Buse J, Krause RD. Clinical implications for biochemical diagnostic thresholds of adrenal sufficiency using a highly specific cortisol immunoassay. Clin Biochem. 2017;50(9):475-80.
8. Mongioi LM, Condorelli RA, Barbagallo F, Cannarella R, La Vignera S, Calogero AE. Accuracy of the Low-Dose ACTH Stimulation Test for Adrenal Insufficiency Diagnosis: A Re-Assessment of the Cut-Off Value. J Clin Med. 2019;8(6).
9. Grassi G, Morelli V, Ceriotti F, Polledri E, Fustinoni S, D'Agostino S, et al. Minding the gap between cortisol levels measured with second-generation assays and current diagnostic thresholds for the diagnosis of adrenal insufficiency: a single-center experience. Hormones (Athens). 2020;19(3):425-31.
